# Experimental evolution reveals habitat-specific fitness dynamics among *Wolbachia* clades in *Drosophila melanogaster*

**DOI:** 10.1111/mec.12643

**Published:** 2014-01-29

**Authors:** Elisabetta Versace, Viola Nolte, Ram Vinay Pandey, Ray Tobler, Christian Schlötterer

**Affiliations:** * Institut für Populationsgenetik, Vetmeduni ViennaVeterinärplatz 1, Wien, 1210, Austria; † Graduate School of Population GeneticsWien, Austria

**Keywords:** *Drosophila melanogaster*, experimental evolution, mtDNA, Pool-Seq, temperature, *Wolbachia pipientis*

## Abstract

The diversity and infection dynamics of the endosymbiont *Wolbachia* can be influenced by many factors, such as transmission rate, cytoplasmic incompatibility, environment, selection and genetic drift. The interplay of these factors in natural populations can result in heterogeneous infection patterns with substantial differences between populations and strains. The causes of these heterogeneities are not yet understood, partly due to the complexity of natural environments. We present experimental evolution as a new approach to study *Wolbachia* infection dynamics in replicate populations exposed to a controlled environment. A natural *Drosophila melanogaster* population infected with strains of *Wolbachia* belonging to different clades evolved in two laboratory environments (hot and cold) for 1.5 years. In both treatments, the rate of *Wolbachia* infection increased until fixation. In the hot environment, the relative frequency of different *Wolbachia* clades remained stable over 37 generations. In the cold environment, however, we observed marked changes in the composition of the *Wolbachia* population: within 15 generations, one *Wolbachia* clade increased more than 50% in frequency, whereas the other two clades decreased in frequency, resulting in the loss of one clade. The frequency change was highly reproducible not only among replicates, but also when flies that evolved for 42 generations in the hot environment were transferred to the cold environment. These results document how environmental factors can affect the composition of *Wolbachia* in *D. melanogaster*. The high reproducibility of the pattern suggests that experimental evolution studies can efficiently determine the functional basis of habitat-specific fitness among *Wolbachia* strains.

## Introduction

*Wolbachia* are intracellular α-proteobacteria that infect many arthropod and nematode species (Werren *et al*. 2008). These bacteria are currently considered the most widespread endosymbionts in arthropods (Hilgenboecker *et al*. 2008; Zug & Hammerstein 2012) and are an important target of research for the control of disease vectors and pests (Hoffmann *et al*. 2011; Walker *et al*. 2011; O'Connor *et al*. 2012; Bian *et al*. 2013). The factors that determine the incidence of *Wolbachia* strains in host populations are not well described, although they are crucial to understand and control the evolutionary dynamics of these bacteria.

The phenotypic effects of *Wolbachia* in *Drosophila melanogaster* include the manipulation of the host reproduction (for instance cytoplasmic incompatibility (CI), see Werren *et al*. (2008)), antiviral protection (Hedges *et al*. 2008; Teixeira *et al*. 2008), improved metabolic processes (Brownlie *et al*. 2009; Ikeya *et al*. 2009; Hosokawa *et al*. 2010) and increased fecundity (e.g. Olsen *et al*. 2001; Fry *et al*. 2004). In *D. melanogaster, Wolbachia* is transmitted vertically from mother to offspring (Richardson *et al*. 2012; Early & Clark 2013; Ilinsky 2013). In nature, the transmission of the infection is not perfect (Hoffmann *et al*. 1998). For this reason to be maintained or spread in the population, *Wolbachia* must manipulate the host reproductive processes in favour of infected flies or confer a fitness advantage to their hosts (Caspari & Watson 1959; Fine 1978; Hoffmann *et al*. 1998). One additional layer of complexity of *Wolbachia* dynamics comes from the diversity of strains in natural populations (e.g. Osborne *et al*. 2009 for *D. simulans*), which were recently grouped into five distinct clades (Richardson *et al*. 2012). Using temporal samples, Riegler *et al*. (2005) showed a turnover of *Wolbachia* clades in the recent past, with the wMel type becoming the most abundant variant. Surveys of *Wolbachia* in natural *D. melanogaster* populations have confirmed the predominance of the derived wMel in recent collections (Nunes *et al*. 2008b; Richardson *et al*. 2012; Early & Clark 2013).

Although the spread of *Wolbachia* in natural *Drosophila* populations can be very fast (Turelli & Hoffmann 1991; Nunes *et al*. 2008b), the rate of infection between different populations is highly variable (Turelli & Hoffmann 1991; Hoffmann *et al*. 1994, 1998; Solignac *et al*. 1994; James & Ballard 2000; Ilinsky & Zakharov 2007; Verspoor & Haddrill 2011). For instance, in 12 natural sites along the East coast of Australia, infection rates of *D. melanogaster* ranged from 18% to 85% (Hoffmann *et al*. 1994), and in 13 natural sites in California, *D. simulans* infection rates varied between 11% and 96% (Turelli & Hoffmann 1991). Because only few studies analysed populations collected at multiple time points (but see Turelli & Hoffmann 1991; Nunes *et al*. 2008b), the interpretation of differences in *Wolbachia* infection rates among populations is not easy. Several factors can explain the prevalence of *Wolbachia* in natural populations such as population history, genetic drift, environmental conditions (e.g. Hoffmann *et al*. 1986; Clancy & Hoffmann 1998; Reynolds & Hoffmann 2002; Jia *et al*. 2009), bacterial density (Mouton *et al*. 2007), *Wolbachia* strain and host genotype (Olsen *et al*. 2001; Fry *et al*. 2004), virus protection properties (Hedges *et al*. 2008; Teixeira *et al*. 2008), transmission rate (Turelli & Hoffmann 1995), cytoplasmic incompatibility (Caspari & Watson 1959), *Wolbachia*-mediated fecundity differences (e.g. Olsen *et al*. 2001; Fry *et al*. 2004) and interaction of these factors. Yet, linking *Wolbachia* dynamics in natural populations to the causative factor(s) is challenging because natural populations are not only exposed to uncontrolled environmental variability, they also differ in *Wolbachia* infection rate and composition. Experimental evolution in controlled environments can be used to overcome these difficulties and to better understand the role of specific environmental factors in the maintenance or spread of *Wolbachia* infection. Moreover, this approach can clarify the habitat-dependent differences of specific strains and clades.

In this work, we use a combination of experimental evolution and next-generation sequencing to investigate the temporal dynamics of *Wolbachia* infection in *D. melanogaster*. A natural-derived population of *D. melanogaster* infected with different *Wolbachia* strains was exposed for subsequent generations to either a cold or a hot temperature regime. We observe that in both environments, the infection rate rapidly increased from 53% to 100%, indicating that *Wolbachia* confers a fitness advantage to infected flies over uninfected flies. To investigate the functional divergence of different *Wolbachia* clades, we traced their relative abundance over time. While in the hot treatment *Wolbachia* clade composition in the population remains stable, in the cold environment *Wolbachia* clades change in their relative frequency. When we expose fully infected populations of hot evolved flies to the cold environment, we observe the same response recorded in cold evolved flies, showing that the repeatability of our findings is independent of the initial genetic background of the host and the presence of uninfected flies. We discuss to what extent our results could be extrapolated to processes in natural populations.

## Materials and methods

### Drosophila melanogaster population and culture conditions

A natural *Drosophila melanogaster* population collected in Northern Portugal (Povoa de Varzim) was maintained as 113 isofemale lines for five generations at laboratory conditions (Orozco-terWengel *et al*. 2012). From the isofemale lines, 10 independent replicate base populations (BP) were established, each with 565 females (five females from each isofemale line per replicate) (Orozco-terWengel *et al*. 2012). Five replicates were exposed to a hot temperature regime (H) that fluctuated between 12 h 18 °C/dark and 12 h 28 °C/light, resulting in a new generation about every 2 weeks. A new generation was set up at the peak of eclosion (0–2 days after the beginning of the eclosion) by transferring 1000 adult flies into new bottles for egg laying. After 2 days of egg laying, flies were transferred to a fresh set of bottles for other 2 days. Eclosing flies from the first egg laying were only used for the next generation if <1000 flies were obtained from the second egg laying. The other five replicates were maintained in a cold environment (C) that fluctuated between 12 h 10 °C/dark and 12 h 20 °C/light, resulting in a new generation about every 4 weeks. New generations were set up in the same way as for the hot regime. After 42 generations, three replicates of the hot evolved flies were shifted to and maintained in the cold environment for 15 generations (HC). Although the only independent variable explicitly manipulated was the temperature regime, the two treatments could differ in other respects that covary with temperature, such as egg density, development time, food deterioration, presence and fitness of different pathogens. The experimental evolution procedure only used nonoverlapping generations. Amplification and culture conditions are further described in Orozco-terWengel *et al*. (2012).

### Sequencing of individual flies from the base population and Pool-Seq of experimental evolution samples

Twelve female flies from the base population (BP replicate 2) were sequenced individually. Table S1 (Supporting information) summarizes the type of reads and average coverage for each sequenced individual. Libraries were prepared following standard protocols using 12 index adapters from the truseq v2 DNA Sample Prep Kit (Illumina, San Diego, CA, USA) and sequenced on a 2 × 100 bp paired-end run.

To monitor the *Wolbachia* composition during the experimental evolution study, we sequenced pools of flies (Pool-Seq) at different time points: three replicates of the base population at the beginning of the experiment (BP, see Orozco-terWengel *et al*. (2012)); four replicates at generation 15 evolved in the cold environment (C15a, C15b, C15c and C15d); two replicates at generation 15 and a third replicate at generation 23 for flies evolved in the hot environment (H23a, H15b and H15c, see Orozco-terWengel *et al*. (2012)); three replicates evolved in the hot environment at generation 37 [H37a, H37b and H37c, see (Orozco-terWengel *et al*. 2012)]; three replicates evolved for 42 generations in the hot environment and then for 15 generations in the cold environment (HC57a, HC57b and HC57c, see Tobler *et al*. (2013)).

Illumina sequencing reads for the base population and hot evolved replicates have already been reported in Orozco-terWengel *et al*. (2012). For the new data, genomic DNA of 500 pooled females from each replicate was extracted as described in Orozco-terWengel *et al*. (2012) or using a high salt extraction protocol (Miller *et al*. 1988). Due to the use of different library preparation protocols during the experiment (Table S2, Supporting information), the starting material of genomic DNA varied between 5 and 1 μg. Genomic DNA was sheared using a Covaris S2 device (Covaris, Inc., Woburn, MA, USA), and paired-end libraries were prepared using the Paired-End DNA Sample Preparation Kit (Illumina) or the NEBNext DNA Sample Prep modules (New England Biolabs, Ipswich, MA, USA) in combination with index adapters from the truseq v2 DNA Sample Prep Kit (Illumina). All libraries were size-selected on agarose gels and amplified using 10 PCR cycles with recommended denaturation, annealing and extension temperatures. Table S2 (Supporting information) summarizes the type of reads, sequencing machine used and average coverage for each sequenced replicate.

### Test of Wolbachia infection status

We determined the *Wolbachia* infection status of individual flies by PCR amplification of the *Wolbachia wsp* gene. We analysed at least 44 adult individuals in the base population (BP), the cold evolved populations generation 15 (C15a and C15b), hot evolved populations generation 15 (H15b and H15c), cold evolved population generation 33 (C33a and C33b), Hot evolved population generation 37 (H37b and H37c) and HotCold evolved population generation 57 (HC57b and HC57c). DNA was extracted from individual flies using a high salt extraction protocol (Miller *et al*. 1988). We used primers wsp81F (5′-TGGTCCAATAAGTGATGAAGA AAC-3′) and wsp691R (5′-AAAAATTAAACGCT ACTCCA-3′) (Braig *et al*. 1998) to amplify a 630-bp fragment of the *Wolbachia wsp* gene. As an internal control for the presence of DNA and for correct PCR conditions, we chose primers crmF (5′-GCTGGACGCAGGCGAATG-3′) and crmR (5′-GGATGTGGGTGGTAGGA GAC-3′) to co-amplify a 709-bp fragment of nuclear *D. melanogaster* DNA in the same reaction. Reactions in which the control primers resulted in an undetectable or very weak band were repeated with an alternative pair of internal control primers (Lhr-1-F: 5′-GGTATCCCTTCCTCATCATCC-3′ and Lhr-1-R: 5-AGCTGTCGAGTGGCTTTCTCT-3′) (Nolte *et al*. 2008) that produce a 466-bp band. PCR was carried out in 20 ml reaction volumes using 2.5 mm MgCl_2_, 0.2 mm dNTPs, 10 pmol of each primer, 1 U FirePol *Taq* Polymerase in buffer B (Solis Biodyne, Tartu, Estonia) and approximately 20 ng genomic DNA. Cycling conditions were as follows: 3 min at 94 °C for initial denaturation followed by 32 cycles of 94 °C per 30 s, 55 °C per 30 s, 72 °C per 50 s and a final extension of 72 °C per 7 min.

We also determined the *Wolbachia* infection of the 12 individually sequenced flies of the BP using their sequenced reads. A fly was considered infected with *Wolbachia* when the consensus sequence covered more than 95% of the *Wolbachia* genome, and the ratio of the mean coverage of the *Wolbachia* and *D. melanogaster* genomes was higher than one. Identical results were obtained when we applied the criteria of Richardson *et al*. (2012). We calculated average coverage from mpileup files using custom Perl scripts.

### Mapping of reads

Before mapping the reads to the reference genome, we trimmed them as described in Kofler *et al*. (2011a) to remove low quality bases. The mapping parameters, filtering for mapping quality and indel masking are described in Orozco-terWengel *et al*. (2012).

Reads of the individual flies were mapped with burrows-wheeler aligner (BWA), version 0.5.8c (Li & Durbin 2009) on a Hadoop cluster with DistMap (Pandey & Schlötterer 2013) against the *D. melanogaster* (version 5.18), *Acetobacter pasteurianus* (AP011121.1), *Lactobacillus brevis* (CP000416.1) and *Wolbachia pipientis* (NC_002978.6) reference genomes. Pool-Seq reads were mapped with BWA version 0.5.7 against the *D. melanogaster* (version 5.18, without the mtDNA sequence contained in the U contig) and *Wolbachia* (NC_002978.6) reference genomes. To use the same mtDNA alignment for the phylogenetic analysis as Richardson *et al*. (2012), we remapped the mtDNA reads against the mtDNA sequence contained in the U chromosome of the *D. melanogaster* genome.

### Consensus sequences and variant calling

From the 12 flies of the base population that were individually sequenced, we generated the consensus sequences of the four *Wolbachia* genomes of the infected flies (w2, w6, w14 and w18) and of the 12 mitochondrial genomes (mt2, mt4, mt5, mt6, mt7, mt12, mt13, mt14, mt15, mt16, mt18 and mt19).

Consensus sequences for the *Wolbachia* and mtDNA genomes were built using varscan release 2 (Koboldt *et al*. 2012). For *Wolbachia* (mtDNA), we used the following parameters: minimum read depth 50 (100), minimum base quality 15 (15) and minimum variant allele frequency threshold 0.5 (0.5). Bases deleted relative to the reference were coded as Ns, and insertions were excluded. Consensus sequences are available in Appendix S1 (Supporting information).

### Phylogenetic analysis

We performed a phylogenetic analysis for *Wolbachia* and mtDNA using our consensus sequences, the reference genomes and 179 genomes described in Richardson *et al*. (2012). After combining all the available sequences, we removed the alignment columns that contained one or more N or ambiguous characters and converted the multiple alignment files into Phylip format using seaview, version 4 (Gouy *et al*. 2010).

We reconstructed the phylogenies with raxml, version 7.4.4 (Stamatakis 2006) using maximum likelihood (ML) with a general time reversible (GTR) model of nucleotide substitution, gamma rate heterogeneity and the rapid bootstrap algorithm (Stamatakis *et al*. 2008). Bootstrap ML trees in Newick format are available in Appendix S2 (Supporting information).

### Identification of clade-specific SNPs and estimate of clade frequency

Clade-specific SNPs for *Wolbachia* and mtDNA (available in the Appendix S3, Supporting information) were identified based on all strains used in the phylogenetic analysis. As clades I, II and III had most variants in common, we grouped them together and identified clade-specific SNPs for clade I_II_III, clade IV, clade V and clade VI. Given the full concordance of mtDNA and *Wolbachia,* we use the same clade identifier for both genomes.

To check whether the clade-specific SNPs captured all relevant *Wolbachia* and mitochondrial diversity present in our experimental populations, we determined the cumulative frequency of all clade-specific variants. As expected, the cumulative frequency was very close to one, indicating that no major variant was missed (Fig. S1, Supporting information). For each sequenced replicate, we estimated the frequency of different *Wolbachia* and mitochondrial lineages based on the clade-specific SNPs. To ensure that coverage differences across replicates were not affecting our analysis, we downsampled the *Wolbachia* data to a 50-fold coverage and the mtDNA data to 100-fold coverage using popoolation2 (Kofler *et al*. 2011b). Similar results were obtained without downsampling (data not shown).

### Estimating the impact of neutral drift on frequency changes of *Wolbachia* clades

We tested whether the change in frequency observed for clade V was more extreme than expected under neutral evolution as follows. The starting frequency for clade V was determined calculating the median of the clade-specific SNP frequencies for each of the three replicates. We then generated the expected distribution of clade frequencies after a given number of generations of neutral evolution, conditional on the starting clade frequency. The end frequency distribution was obtained by raising an *N *+* *1 by *N *+* *1 Markovian transition matrix to the *n*th power, where *N* is the effective population size, *n* is the number of generations and the cell in the *i*th row and *j*th column is the binomial probability *P*(*j* | *i*/*N*). The final frequency distribution of a clade with starting frequency *i*/*N* is the *i*th + 1 row of the resulting matrix. The probability of the observed final allele frequency was then taken as 2× the probability of the observed end frequency of clade V given the final frequency distribution, based on a 2-sided alternative hypothesis. We used an effective population size of 75 for our simulations based on the estimated *N* from Tobler *et al*. (2013). Because all three replicates were independent, we multiplied the *P*-values from the three replicates to obtain the probability that the observed frequency change could be attributed to genetic drift. Note that our test does not account for the increase in *Wolbachia* infection rate during the experiment.

### Estimation of selection coefficient

For simplicity, we assumed that clade V has the same fitness advantage relative to each of the other clades present in the population. We determined the starting and end frequency of clade V by the average frequency of the clade-specific SNPs in each of the three replicates. The selection coefficient *s* was obtained from:
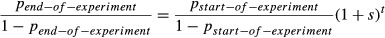


with *p* being the frequency of clade V and *t* the number of generations.

## Results

### Rate of Wolbachia infection

We determined the *Wolbachia* infection rate with an individual-based PCR assay in the base population (BP) and in the evolved populations at different time points (Table 1). In both temperature environments the infection rate increased, and in less than 37 generations the *Wolbachia* infection was fixed in the population. While it has been previously suggested that fixation of *Wolbachia* is expected in laboratory cultures due to the high transmission rate and cytoplasmic incompatibility (e.g. Friberg *et al*. 2011), this is, to our knowledge, the first documented increase in infection rate of *Drosophila melanogaster* populations evolving in the laboratory. It is not clear whether the fast spread of *Wolbachia* infection has been caused by CI through an increase in the fitness of infected females *versus* noninfected females or by fitness advantages mediated by protection against viral infection (Hedges *et al*. 2008; Teixeira *et al*. 2008), improved metabolic processes (Brownlie *et al*. 2009; Ikeya *et al*. 2009; Hosokawa *et al*. 2010) or increased fecundity of the *D. melanogaster* host (e.g. Olsen *et al*. 2001; Fry *et al*. 2004). These factors in fact can produce similar selection effects.

**Table 1:** *Wolbachia* infection rate at different time points

Screened replicate	Screened flies	% infected flies
BP	44	53
C15 replicate a	48	60
C15 replicate b	48	92
H15 replicate b	48	88
H15 replicate c	48	71
C33 replicate a	48	100
C33 replicate b	48	100
H37 replicate b	48	100
H37 replicate c	48	100
HC57 replicate c	48	100

BP, Base population; C15, Cold evolved generation 15; H15, Hot evolved generation 15; C33, Cold evolved generation 33; H37, Hot evolved generation 37; HC57, HotCold evolved generation 57.

### Wolbachia diversity in the base population

We used sequencing of individual flies to obtain an overview of the *Wolbachia* diversity in the base population in our experiment. In total, 12 *D. melanogaster* females were sequenced, and the *Wolbachia* and *D. melanogaster* mtDNA genomes were assembled by mapping reads onto reference genomes (Table 2, Appendix S1, Supporting information for the consensus sequences). The average coverage for the infected (uninfected) flies is 23.80 ± 10.95 (26.07 ± 4.46) for the nuclear genome, 189.95 ± 64.28 (3.07 ± 0.39) for the *Wolbachia* genome and 3125.71 ± 2343.37 (2742.41 ± 828.90) for the mitochondrial genome. We classified the obtained sequences through phylogenetic analysis in combination with previously published *Wolbachia* and mtDNA genomes of *D. melanogaster* (Wu *et al*. 2004; Richardson *et al*. 2012).

**Table 2:** Sequencing of *Drosophila melanogaster* individuals

Strain	*Wolbachia* clade	*Wolbachia* alternative nomenclature based on Ilinsky (2013)	mtDNA clade	Average coverage *Wolbachia*	Average coverage nuclear *D. melanogaster* genome	*Wolbachia*/*D. melanogaster* nuclear coverage
w2	Clade VI	wMelCS	Clade VI	209.91	17.07	12.30
w4	n.i.	wMel	Clade VI	3.44	24.76	0.14
w5	n.i.	wMel	Clade III	2.88	24.01	0.12
w6	Clade VI	wMelCS	Clade VI	139.10	16.69	8.34
w7	n.i.	wMel	Clade VI	2.71	20.90	0.13
w12	n.i.	wMel	Clade III	3.23	24.94	0.13
w13	n.i.	wMel	Clade VI	3.75	33.20	0.12
w14	Clade I	wMel	Clade I	272.23	39.89	6.82
w15	n.i.	wMel	Clade I	2.67	25.66	0.10
w16	n.i.	wMel	Clade V	2.727	22.56	0.12
w18	Clade V	wMel	Clade V	138.56	21.57	6.42
w19	n.i.	wMel	Clade I	3.23	32.57	0.10

n.i., not infected.

Our analysis showed that the four *Wolbachia* strains belong to three different clades: w2 and w6 strains group to clade VI and the w14 strain to clade I (Fig. S2A, Supporting information). The *Wolbachia* strain w18 did not cluster with any of the *Wolbachia* clades present in the phylogenetic tree shown by Richardson *et al*. (2012). The corresponding mtDNA, mt18, clustered instead to clade V. A complete congruence of mtDNA and *Wolbachia* genealogies has been widely documented in *D. melanogaster* (Richardson *et al*. 2012; Early & Clark 2013; Ilinsky 2013). On this basis, we could assign the *Wolbachia* strain w18 to clade V, the clade of its corresponding mtDNA (Fig. S2A, Supporting information). To our knowledge, this is the first complete genome assembled for *Wolbachia* clade V. The mtDNA sequences of the uninfected flies belonged to the same clades as the infected ones, with the exception of mtDNA12 and mtDNA5, which mapped to clade III (we found no infected flies in clade III).

We measured the relative frequency of the different *Wolbachia* and mtDNA clades in the Pool-Seq data from the base populations by calculating the median frequency of the SNPs private to each clade (Appendix S4, Supporting information). Clade VI was most common for *Wolbachia* (∼55%), followed by clade V (∼25%) and clades I_II_III (∼20%). Clade IV was not detected. The relative mtDNA frequencies were similar, but clade V (∼ 36%) was slightly more frequent than clade VI (∼30%). The high frequencies of clades V and VI contrast previous results (Riegler *et al*. 2005; Nunes *et al*. 2008b; Richardson *et al*. 2012), which did not detect *Wolbachia* from clade V and found only a few flies infected with clade VI. While clade V seems to be widely distributed in European *D. melanogaster* (Ilinsky 2013), although not in all populations (Early & Clark 2013), the high frequency of clade VI is, to our knowledge, unprecedented.

### Response of Wolbachia haplotypes to cold and hot environments

Fitness differences among *Wolbachia* lineages can be inferred by monitoring their relative frequency changes over time. We measured the relative frequency changes during the experimental evolution study by tracking the median frequency of clade-specific SNPs (single nucleotide polymorphisms) (Appendix S4, Supporting information). Because strains from clades I, II, and III were only slightly diverged, we treated them as a single group (clade I_II_III) to have a higher confidence in our allele frequency estimates.

**Fig 1:**
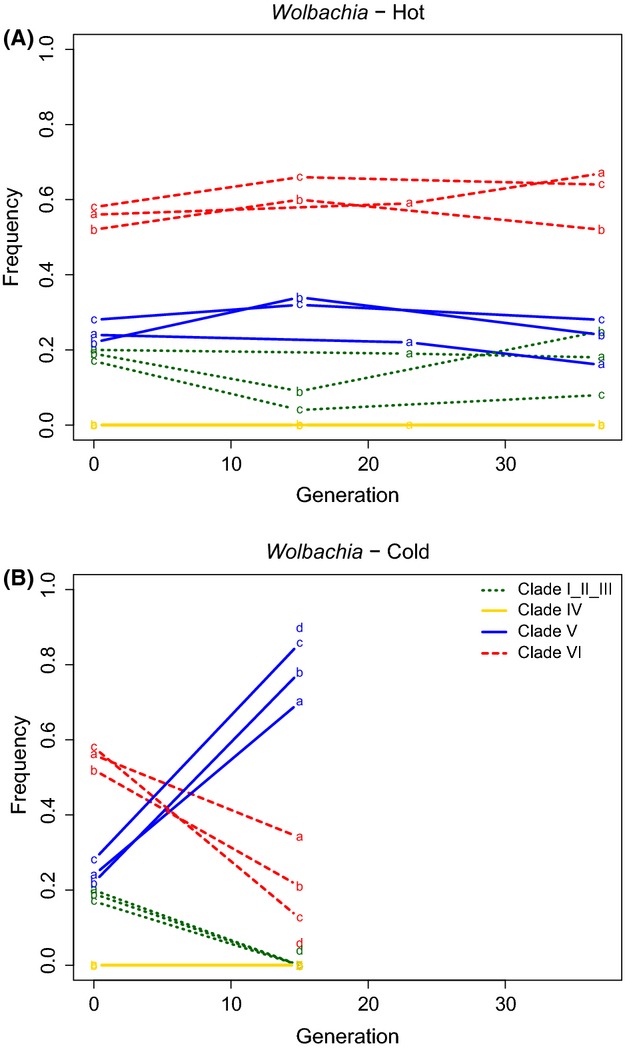
Trajectories of *Wolbachia* clades in hot and cold laboratory environments. We used the median frequency of clade-specific SNPs to estimate the relative clade frequency. (A) The relative frequencies of different *Wolbachia* clades over 37 generations of evolution in the hot environment (fluctuating between 18 and 28 °C). (B) The relative frequencies of *Wolbachia* clades over 15 generations in the cold environment (fluctuating between 10 and 20 °C). Each letter indicates a replicate population and * marks significant comparisons (*P* < 0.05, after Bonferroni correction). While in the hot environment the relative frequency of the *Wolbachia* clades remains relatively stable, clade V increased by about 50% in the cold environment.

In the hot environment, the *Wolbachia* composition (i.e. diversity) remained stable over 37 generations (Fig.1A), with clade I_II_III being at the lowest (∼20%), clade V at intermediate (∼25%) and clade VI at the highest frequency (∼55%). The long-term stability of the three *Wolbachia* clades suggests that they either have similar fitness in the hot environment or that their frequency is stabilized by selection. More generations of experimental evolution would be needed to distinguish between these two scenarios.

In the cold environment, however, the relative frequency of strains from the three clades changed substantially within 15 generations, and the changes were highly consistent across replicates (Fig.1B). As a result, clade I_II_III was almost completely lost (lost in three replicates and present at 4% in one replicate), clade VI dropped from ∼55% to ∼20% and clade V became the most abundant after a frequency increase of almost 55% (from ∼25 to ∼80%). The consistent changes in frequency of the different clades across all three replicates, in combination with the stability in the hot environment, suggest that the frequency changes in the cold environment are not caused by random genetic drift. We tested the probability of genetic drift causing the frequency change of clade V for the cold and hot environment. Tobler *et al*. (2013) estimated an effective population size (*N*) of about 300 chromosomes for both temperature regimes. Assuming an *N* of 75 for our simulations of *Wolbachia* responses, the probability of observing a frequency change at least as pronounced as in our experiment was <4.3 × 10^−6^ for the cold environment and 0.36 for the hot environment. Assuming that clade V has the same selective advantage relative to the other clades, the inferred selection coefficient *s* for generation 15 ranged from 0.14 to 0.2 in the three cold replicates.

While fitness differences are the most likely explanation for the dynamics in the cold environment, based on the relative frequencies of *Wolbachia* clades alone it is not clear whether mtDNA, *D. melanogaster* or *Wolbachia* are the drivers of this pattern.

### Response of mtDNA types in cold and hot environments

Our sequencing of individual flies already indicated that all but one of the mtDNA clades found in our starting population are represented either in flies infected with *Wolbachia* or uninfected flies (clade III is not associated with *Wolbachia* infection). Given that the base population had an infection rate of approximately 50% and that the infection is fixed by generation 37, a comparison between the frequency changes of mtDNA and *Wolbachia* can shed light on the target of selection. If selection operates on mtDNA, we expect the same frequency change for mtDNA and *Wolbachia*. On the other hand, if *Wolbachia* is the target of selection, we expect a more pronounced frequency change for *Wolbachia*. This difference arises from the presence of uninfected flies in the population. In fact, while frequency changes for *Wolbachia* are determined from infected flies only, mtDNA frequencies are based on infected and uninfected flies.

**Fig 2:**
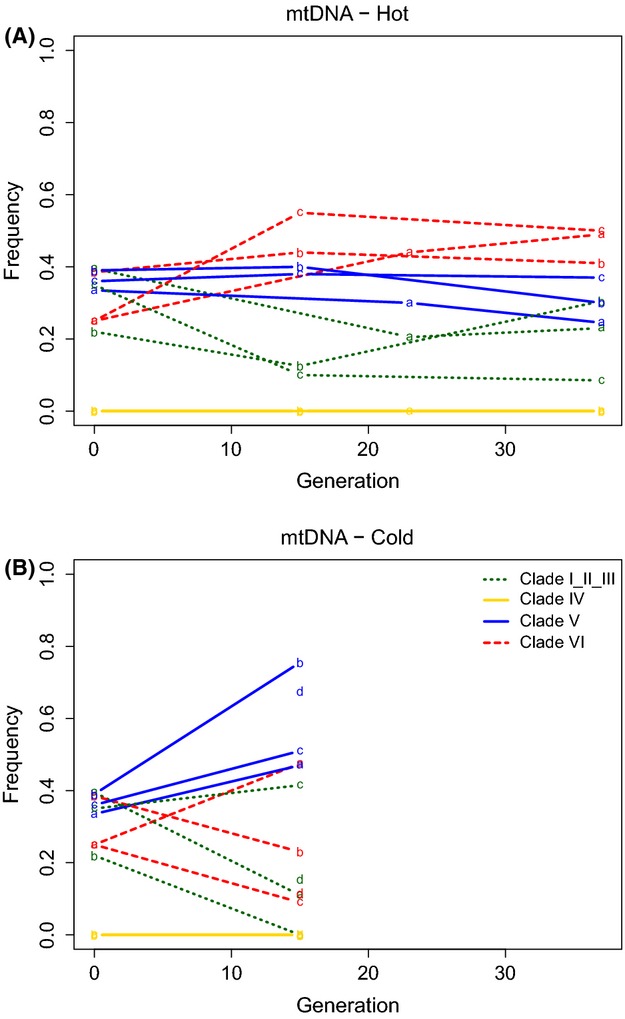
Trajectories of mtDNA clades in hot and cold laboratory environments. We used the median frequency of clade-specific SNPs to estimate the relative clade frequency. The relative frequencies of mtDNA clades in the hot (A) and cold (B) environment are shown. Despite being more variable than what we observe for *Wolbachia*, also the mtDNA trajectories show little changes in clade composition in the hot environment and a pronounced frequency increase for mtDNA clade V. Each letter indicates a different population replicate.

Overall, we found the patterns of mtDNA dynamics to be highly similar to those observed for *Wolbachia* (Fig.2). But before generation 37, when the population is not fully infected with *Wolbachia*, we observe some differences between *Wolbachia* and mtDNA responses in terms of relative frequency changes. In analogy with the pattern described in *Wolbachia*, in the hot environment we observe only moderate frequency changes for mtDNA. In the cold environment clade V increased in frequency (up to 30%) but this increase was not as strong as in the corresponding *Wolbachia* clade. Hence we can conclude that the observed frequency changes are not driven by fitness differences of mtDNA haplotypes.

### The frequency change in the cold environment does not depend on the initial genetic background of the host or the presence of uninfected flies

**Fig 3:**
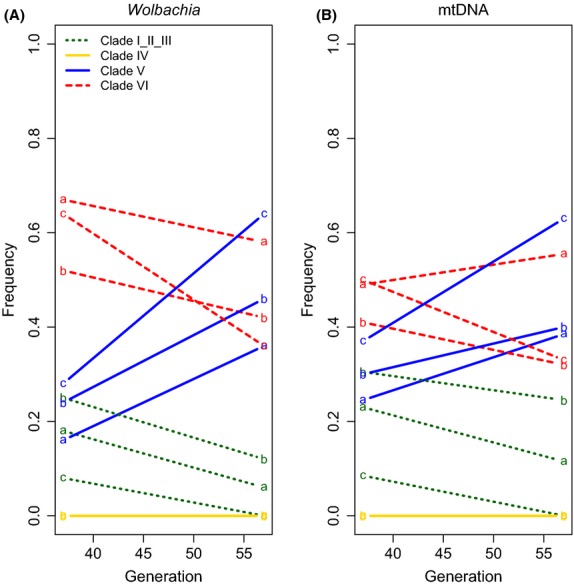
Trajectories of *Wolbachia* and mtDNA clades after shifting hot evolved flies to the cold environment. At generation 42, flies were moved from the hot environment to the cold one. The frequency change from generation 37 in the hot environment to generation 57, with 15 generations in the cold environment is shown. The relative frequency of the different clades for *Wolbachia* (A) and mtDNA (B) is shown. Both *Wolbachia* and mtDNA experience a marked increase in frequency of clade V. Replicate populations are indicated by small letters.

While the previous analyses pointed towards *Wolbachia* as the driver of the frequency changes in the cold environment, it is still possible that the host genotype also contributed to the observed pattern. We performed a further experiment to distinguish between host and *Wolbachia*-mediated effects in the cold environment. We shifted flies that evolved in the hot environment for 42 generations to the cold environment for 15 additional generations. We then compared the relative frequency of different clades between generation 37 (H37) and generation 57 (HC57). We then compared the relative frequency of different clades between generation 37 (H37) and generation 57 (HC57), both with a 100% *Wolbachia* infection rate. Interestingly, the trajectories for the three *Wolbachia* clades closely resemble those observed in the BP-C15 comparison (Fig.3). Clade V increases again substantially in frequency at the expense of clade I_II_III and VI. As for the frequency change at previous time points, we used forward Wright–Fisher simulations to determine the probability that the frequency change of clade V could be explained by drift. Again, we find that drift alone is highly unlikely to explain the parallel frequency change of clade V in the three replicates (*P* = 0.014). Importantly, all flies were infected with *Wolbachia*, which allows for a more precise *N* estimate than in the BC comparison, for which we did not account for the increase in infection rate during the experiment. Assuming that clade V has the same selective advantage relative to the other clades, the inferred selection coefficient *s* ranged from 0.068 to 0.11 in the three replicates.

Apart from providing further evidence for nonrandom changes in *Wolbachia* composition in the cold environment, this result also allows some conclusions about the influence of the host genotypes. Random mating shuffles the nuclear host genome with respect to *Wolbachia* and mtDNA genotypes. If the initial host genome had a strong influence on the frequency changes seen in the cold environment, different trajectories would be expected for experiments starting from the initial population or the hot evolved (H42) replicates. Given the very similar trajectories of the *Wolbachia* clades in both experiments, we conclude that the differential fitness effects of different strains in the hot and cold environment are mainly due to the properties of specific *Wolbachia* strains.

One additional important insight into the dynamics in the cold environment is provided by the fact that all flies were infected with *Wolbachia* at the time point when they were transferred from the hot environment to the cold environment. The overall similarity in *Wolbachia* dynamics in partially and fully infected *D. melanogaster* populations suggests that CI between infected and uninfected flies cannot explain the different dynamics of the *Wolbachia* clades.

### Temperature effect on Wolbachia and mtDNA titer

**Fig 4:**
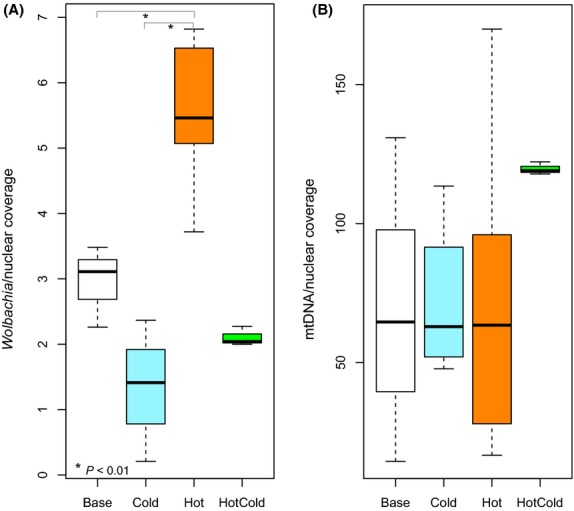
Copy number estimates for *Wolbachia* and mtDNA. We estimated the copy number of *Wolbachia* (A) and mtDNA (B) relative to the nuclear genome using the average read coverage. Despite some heterogeneity among replicates, *Wolbachia* in the hot environment had a higher copy number than in the cold environment. Similarly, flies shifted from the hot to the cold environment (HC) also showed a lower copy number. The base population, which was set up at room temperature, had an intermediate copy number. No significant difference was found for mtDNA. For the HC data, a higher number of mtDNA reads was observed. This is unlikely due to a higher copy number, but is rather the result of a different library kit, which results in a higher coverage of AT rich sequences (see Table S2, Supporting information).

We tested the influence of temperature on the titer of *Wolbachia* and mtDNA, because clade-specific *Wolbachia* titer could in principle produce a change in clade frequency. To account for different read depth among populations, we normalized the average coverage of *Wolbachia* or the mtDNA relative to average coverage of nuclear DNA. Because even populations maintained at the same temperature were not always of the same age when the flies were frozen, we expected some heterogeneity among replicates. Nevertheless, *Wolbachia* in the hot environment had a significantly higher copy number than populations maintained in the cold environment (Fig.4A). This influence of temperature on *Wolbachia* density has been noted before (Clancy & Hoffmann 1998; Hurst *et al*. 2000; Mouton *et al*. 2006; Correa & Ballard 2012). For the mtDNA, no difference was noted among different temperature regimes (Fig.4B).

One important limitation of Pool-Seq for the inference of *Wolbachia*/mtDNA dynamics is the implicit assumption that the titer of *Wolbachia* and mtDNA does not vary among flies. Our results indicate, however, a high variability among replicates and a systematic difference in titer due to the environment (Fig.4, Table S3, Supporting information).

In principle, the difference in titer between hot and cold environment might affect *Wolbachia* clades in different ways and in turn cause the clade-specific responses observed in the cold environment. Nevertheless, because *Wolbachia* and mtDNA are both transmitted maternally, the concordance between both molecules indicates the extent to which variation in titer poses a problem for the analysis of Pool-Seq data. Given that in H37 and HC57, the *Wolbachia* infection is fixed, we compared the dynamics of the mtDNA and *Wolbachia* clades between generation H37 and HC57 to determine whether titer variation affects our interpretation of the results. Interestingly, we noticed that the frequency changes of the three different clades are very similar. Hence, we conclude that the inference of *Wolbachia* dynamics is robust and not the result of titer heterogeneity.

## Discussion

Global surveys of mtDNA variation in *Drosophila melanogaster* show that European *D. melanogaster* populations harbour more divergent haplotypes than other populations studied (Nunes *et al*. 2008a; Nunes *et al*. 2008b). Although not all European populations of *D. melanogaster* host different *Wolbachia* clades (Early & Clark 2013), we detected a high diversity of *Wolbachia* in our Portuguese population. We identified *Wolbachia* haplotypes from three different clades, including, to our knowledge, the first full genome of clade V. To date, clade V had only been observed in the analysis of mtDNA data of populations from Eurasia (Richardson *et al*. 2012; Early & Clark 2013; Ilinsky 2013). Clades V and VI, which are at low frequency in North America and some European populations (Riegler *et al*. 2005; Nunes *et al*. 2008b; Richardson *et al*. 2012), possibly due to a recent replacement (Riegler *et al*. 2005; Richardson *et al*. 2012), are the most abundant haplotypes in the Portuguese population studied here. The most recently derived haplotypes of our population, which belong to clade I_II_III, are likely to have an Afrotropical ancestry (Richardson *et al*. 2012), whereas the other originate from colder climates.

The presence of multiple *Wolbachia* clades in our starting population is consistent with the high mtDNA divergence in European populations of *D. melanogaster* (Hale & Singh 1991; Nunes *et al*. 2008a). The pronounced variation in *Wolbachia* composition among natural *D. melanogaster* populations can either derive from the evolutionary response of *Wolbachia* to different environments or reflect the evolutionary history of their hosts. Comparative studies of *Wolbachia* in the wild face several challenges: it is hard to find *Drosophila* populations with the same *Wolbachia* composition in regions with different environments, and the environmental complexity makes it difficult to identify the causative environmental variable. Therefore, we used experimental evolution in the laboratory to investigate the infection dynamics of different *Wolbachia* haplotypes at two temperature regimes. In either the hot and cold treatment, *Wolbachia* rapidly went to fixation (in less than 37 generations), showing that in our setting infected flies have a selective advantage over uninfected flies. This fast response can be due to an advantage conferred to the host – for instance, protection against viruses (Hedges *et al*. 2008; Teixeira *et al*. 2008), improved metabolic processes (Brownlie *et al*. 2009; Ikeya *et al*. 2009; Hosokawa *et al*. 2010), increased fecundity (Olsen *et al*. 2001; Fry *et al*. 2004) or survival (Fry *et al*. 2004). *Wolbachia* fixation can also be explained by CI (e.g. Hoffmann 1988; Reynolds & Hoffmann 2002), accompanied with higher transmission fidelity under laboratory conditions (e.g. Friberg *et al*. 2011). Further experiments are needed to identify the contribution of CI, fitness effects on the host and their interactions in explaining the rapid fixation of *Wolbachia* in both hot and cold environment. As CI is a frequency-dependent phenomenon, in which the spread of infected hosts is more rapid at intermediate infection frequencies (Fine 1978), experiments with different starting frequencies could be used to distinguish CI from other selective advantages.

One likely explanation for the increase in *Wolbachia* infection rates in the laboratory is an age structure different from that in natural populations. In our setting, adults are allowed to lay eggs only a few days after eclosion and are then discarded (nonoverlapping generations). Hence, males are considerably younger than in natural populations. In combination with the reported strong expression of CI in young *D. melanogaster* males (Reynolds & Hoffmann 2002; Fry *et al*. 2004; but see Yamada *et al*. 2007), this could explain the lower infection rates in natural populations (e.g. Hoffmann *et al*. 1994; Solignac *et al*. 1994; Nunes *et al*. 2008b; Richardson *et al*. 2012; Ilinsky 2013).

Comparing different clades, we observed marked differences in fitness in the hot and cold environment. While in the hot environment the clade composition of the *Wolbachia* population remained constant over 37 generations, in the cold environment we observed a highly dynamic turnover, with strains from *Wolbachia* clades I_II_III being lost and strains from clade VI being reduced in frequency, while clade V experienced a dramatic increase in frequency (∼50%) in 15 generations. Only haplotypes of clade I_II_III, which were lost in the cold environment, originated from the warm climates of Afrotropics (Richardson *et al*. 2012). Haplotypes that persisted or took over in the cold environment originated in colder Eurasian climates (Richardson *et al*. 2012). This pattern suggests that clades V and VI haplotypes may be better adapted to cold climates.

We excluded the possibility that the highly consistent responses observed were primarily caused by the mtDNA as we observed a stronger response for *Wolbachia* than for mtDNA when a part of the population was not infected. This scenario is consistent only with *Wolbachia* being the driver of the frequency change. In fact, as long as a fraction of the *D. melanogaster* population is still uninfected, any fitness advantage mediated by *Wolbachia* could result in the faster frequency increase in clade V.

Previously, it has been shown that temperature can modulate the strength of CI at different ages in *D. melanogaster* (Reynolds & Hoffmann 2002). Clade-specific effect of temperature on CI may result in clade dynamics similar to those observed in our study. We caution that unidirectional CI (CI between infected and uninfected flies) can hardly account for the dynamics of the *Wolbachia* clades in the cold environment because we saw similar clade dynamics when the infection is fixed (H37-HC57). If CI is involved, it could be temperature-dependent bidirectional CI (interaction between flies infected with different *Wolbachia* clades). Yet, to our knowledge bidirectional CI has not been shown in *D. melanogaster* (but see O'Neill & Karr 1990; James & Ballard 2000 for *D. simulans*).

The egg hatchability, which reflects the strength of CI, has been shown to differ among *Wolbachia*-infected flies, but this effect was attributed to the *D. melanogaster* host, rather than to differences among *Wolbachia* strains (Reynolds & Hoffmann 2002; Fry *et al*. 2004). Host-dependent effects of *Wolbachia* infection have been shown also for fecundity in *D. melanogaster* (Olsen *et al*. 2001; Fry *et al*. 2004). Importantly, our data suggest that the temperature-specific *Wolbachia* dynamics observed in our study cannot be attributed to differences in genetic background, because the response of the initial population after 15 generations was similar to the response of replicates evolved for 37 generations in the hot environment and then moved to the cold environment for 15 more generations. We also excluded that the differences between clades can be accounted by differences in *Wolbachia* titer among clades by noticing that when the population was fully infected with *Wolbachia,* the frequency changes of mtDNA and *Wolbachia* matched extremely well for all clades. Given this evidence, we conclude that temperature can influence the evolutionary dynamics of *Wolbachia* in *D. melanogaster*. In particular, haplotypes originated in warm climates (clade I_II_III) seem to have a lower fitness in cold temperatures than haplotypes originated in colder climates (clade V and clade VI).

The contrasting dynamics of *Wolbachia* strains exposed to different environments could potentially have broader implications. Recent work that has demonstrated the ability of *Wolbachia* to block pathogen transmission holds great promise to control human pathogens, such as dengue virus (Hoffmann *et al*. 2011; Walker *et al*. 2011) and *Plasmodium falciparum* (Bian *et al*. 2013). If the environment affects key parameters of specific strains, such as transmission rate or CI, then environmental factors and strain effects may need to be accounted for to assure the success of these projects.

Our work shows the power of the reduced environmental complexity in experimental evolution studies to investigate ecologically relevant parameters using laboratory conditions. Nevertheless, we are aware that experimental evolution studies cannot fully reflect the dynamics of natural environments. One clear example for this is the increase in *Wolbachia* infection observed in our experimental populations and in other laboratory experiments (e.g. Friberg *et al*. 2011), that is not common in natural populations. While we focused on temperature, we propose that this approach is well suited to dissect other ecological parameters, such as viral infections, population densities and other stress factors possibly influencing the highly dynamic infection patterns of *Wolbachia* in natural populations (Turelli & Hoffmann 1991; Riegler *et al*. 2005; Nunes *et al*. 2008b). Furthermore, the possibility to compete different *Wolbachia* and host genotypes in specific environments provides an excellent opportunity to obtain the functional link between genotype and phenotype and thereby uncovering the functional basis of habitat-specific fitness among *Wolbachia* strains and host-symbiont interactions.

## Data accessibility

Data used for this work are available at the European Sequence Read Archive (http://www.ebi.ac.uk/ena/about/search_and_browse) with Accession numbers ERS362602-ERS362617 and ERS362925-ERS362940. The consensus sequences of the *Wolbachia* and mtDNA sequences are available under the Accession numbers ERS362618-ERS362621 and ERS362941-ERS362952, respectively, and are also available in Appendix S1 (Supporting information).

Bootstrap ML trees in Newick format are available in the Appendix S2 (Supporting information).

Clade-specific SNPs for Wolbachia and mtDNA are available in the Appendix S3 (Supporting information).

Median frequency of SNPs private to each clade is available in the Appendix S4 (Supporting information).

Custom scripts available as Supplementary Material.

## Notes

C.S. conceived the project. V.N. collected the data. E.V. analysed the data. R.V.P. and R.T. contributed to analysis tools. E.V., V.N. and C.S. wrote the paper.

## Supporting Information

Supplementary **Appendix S1** **Appendix S2** **Appendix S3** **Appendix S4** **Table S1** Details about the individual flies sequenced. **Table S2** Details about the populations analysed with Pool-Seq. **Table S3** Influence of temperature on mtDNA and *Wolbachia* coverage. **Fig. S1** Cumulative coverage for clade-specific SNPs. **Fig. S2** Phylogenetic relationship of *Wolbachia* and mtDNA haplotypes.
