# Supplementary material for: Experimental evolution reveals habitat-specific fitness dynamics among Wolbachia clades in Drosophila melanogaster
Source: Mol Ecol. 2014 Jan 29;23(4):802–14. doi: 10.1111/mec.12643 (PMC4260678; doi:10.1111/mec.12643)

Supplementary material

Table S1. Details about the individual flies sequenced

Fly	Read length (paired-end)	Average nuclear coverage*	Average mtDNA coverage	Average *Wolbachia* coverage	Library preparation
2	2x100 bp	17	1993	210	TruSeq DNA Sample Prep v2
4	2x100 bp	25	2689	3	TruSeq DNA Sample Prep v2
5	2x100 bp	24	2125	3	TruSeq DNA Sample Prep v2
6	2x100 bp	16	1709	129	TruSeq DNA Sample Prep v2
7	2x100 bp	21	1416	3	TruSeq DNA Sample Prep v2
12	2x100 bp	25	2316	3	TruSeq DNA Sample Prep v2
13	2x100 bp	33	3727	4	TruSeq DNA Sample Prep v2
14	2x100 bp	40	6635	272	TruSeq DNA Sample Prep v2
15	2x100 bp	26	2879	3	TruSeq DNA Sample Prep v2
16	2x100 bp	23	2839	3	TruSeq DNA Sample Prep v2
18	2x100 bp	22	2165	139	TruSeq DNA Sample Prep v2
19	2x100 bp	33	3957	3	TruSeq DNA Sample Prep v2

* Average coverage based on the chromosomal contigs X, XHet, 2L, 2LHet, 2R, 2RHet, 3L, 3LHet, 3R, 3RHet, 4.

Table S2. Details about the populations analyzed with Pool-Seq

Replicate	Read length/ read type (Paired end=PE, Single end=SE	Average nuclear coverage*	Average mtDNA coverage	Average *Wolbachia* coverage	Library preparation
Base population replicate a	2x101 PE<br>2x151 PE	75	4851	261	Paired-End Sample Prep
Base population replicate b	2x101 PE	35	4541	108	Paired-End Sample Prep
Base population replicate c	2x74 PE<br>76 SE	56	808	126	Paired-End Sample Prep
Hot generation 23 replicate a	2x76 PE	42	699	214	Paired-End Sample Prep
Hot generation 15 replicate b	2x76 PE<br>2x101 PE	58	3345	296	Paired-End Sample Prep
Hot generation 15 replicate c	2x76 PE	47	1320	175	Paired-End Sample Prep
Cold generation 15 replicate a	2x101 PE	79	4440	116	Paired-End Sample Prep
Cold generation 15 replicate b	2x101 PE	89	4243	210	Paired-End Sample Prep
Cold generation 15 replicate c	2x101 PE	62	4303	13	Paired-End Sample Prep
Cold generation 15 replicate d	2x100 PE	62	7066	84	TruSeq DNA Sample Prep v2
Hot generation 37 replicate a	2x101 PE	33	2289	215	Paired-End Sample Prep
Hot generation 37 replicate b	2x101 PE	34	5830	234	Paired-End Sample Prep
Hot generation 37 replicate c	2x101 PE	34	3218	195	Paired-End Sample Prep
HotCold generation 57 replicate a	2x100 PE	41	4875	82	TruSeq DNA Sample Prep v2
HotCold generation 57 replicate b	2x100 PE	61	7604	127	TruSeq DNA Sample Prep v2
HotCold generation 57 replicate c	2x100 PE	55	6482	125	TruSeq DNA Sample Prep v2

* Average coverage based on chromosomal contigs X, XHet, 2L, 2LHet, 2R, 2RHet, 3L, 3LHet, 3R, 3RHet, 4.

Table S3. Influence of temperature on mtDNA and *Wolbachia* coverage

Comparison	*Wolbachia*/nuclear coverage	mtDNA/nuclear coverage
Base population *vs*. Cold	t=2.8019, df=5, p-value=0.03791<br>M_{Base}=2.95, M_{Cold}=1.35	t=-0.0487, df=2.757, p-value=0.9645<br>M_{Base}=69.99, M_{Cold}=71.78
Base population *vs*. Hot	t=-4.3648, df=6.717, p-value=0.003635 *<br>M_{Base}=2.95, M_{Hot}=5.51	t=-0.072, df=3.896, p-value=0.9462<br>M_{Base}=69.99, M_{Hot}=72.91
Base population *vs*. HotCold	t=2.282, df=2.223, p-value=0.1372<br>M_{Base}=2.95, $M_{HotCold}$=2.10	t=-1.4733, df=2.006, p-value=0.2782<br>M_{Base}= 69.99, $M_{HotCold}$=119.68
Cold *vs*. Hot	t=-6.4992, df=7.652, p-value=0.0002285 *<br>M_{Cold}=1.35, M_{Hot}=5.51	t=-0.0421, df=7.78, p-value=0.9674<br>M_{Cold}=71.78, M_{Hot}=72.91
Cold *vs*. HotCold	t=-1.6725, df=3.221, p-value=0.1867<br>M_{Cold}=1.35, $M_{HotCold}$=2.10	t=-3.2674, df=3.048, p-value=0.04581<br>M_{Cold}=71.78, $M_{HotCold}$=119.69

* significant with alpha=0.008 (alpha=0.05 with Bonferroni correction for 6 comparisons)

Figure S1: Cumulative coverage for clade specific SNPs

We plotted the sum of the average frequency for each clade (based on the average frequency of the clade private SNPs) for *Wolbachia* (A) and mtDNA (B). Each color indicates a different population sample. Since the sum was very close to one for each sample, we conclude that no major haplotype was missed in our analysis.

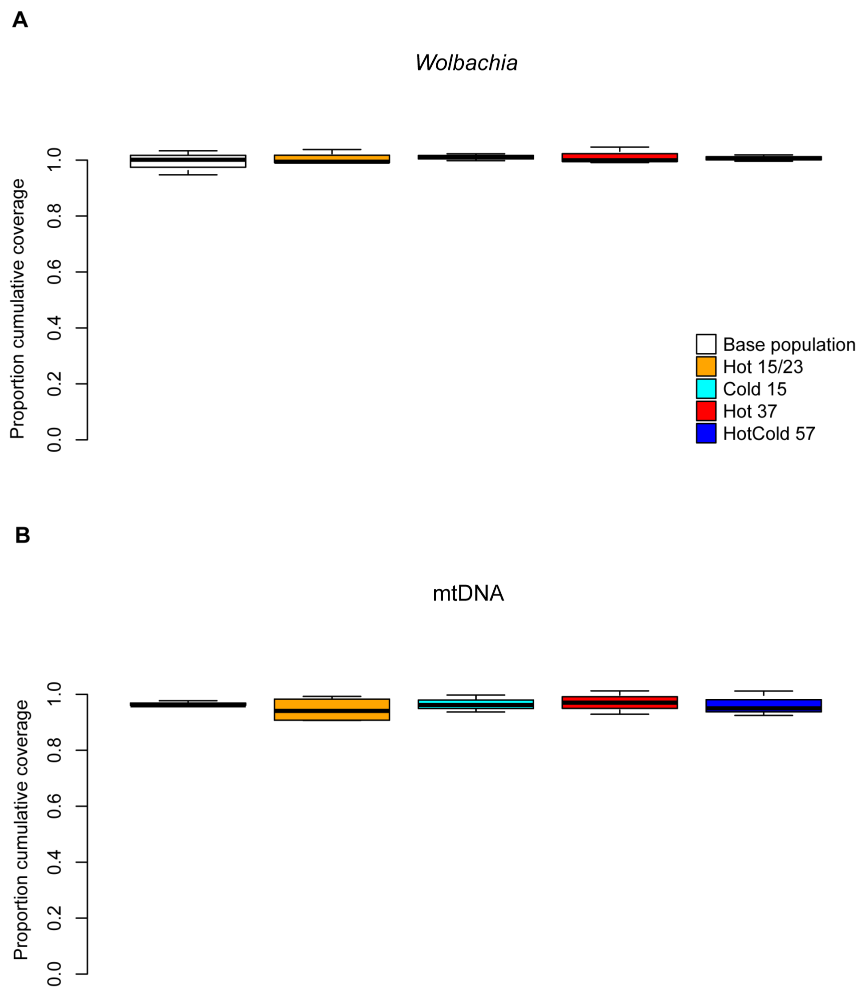

Figure S2: Phylogenetic relationship of *Wolbachia* and mtDNA haplotypes

Maximum likelihood genealogies of *Wolbachia* (A) and mtDNA (B). The trees are based on ungapped multiple alignments of unambiguous bases and midpoint rooting. Bootstrap values are reported for the nodes supporting the different clades. Different colors of the branches indicate grouping of haplotypes used in our analyses. Reference genomes are highlighted in grey. Sequences obtained in this study are indicated by a colored bar.

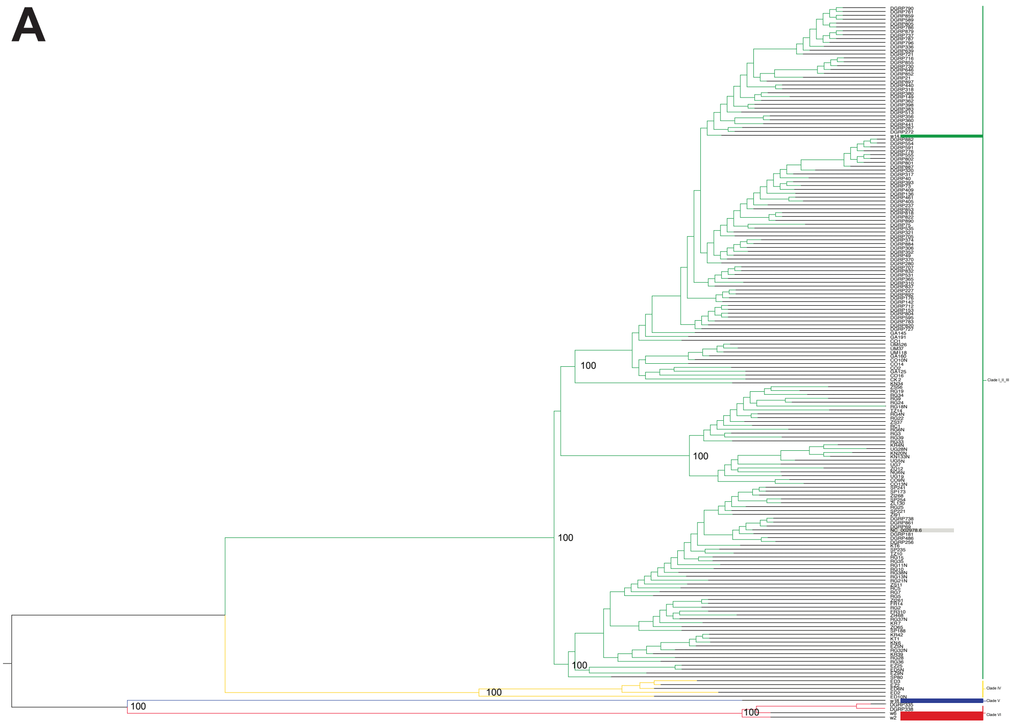

B

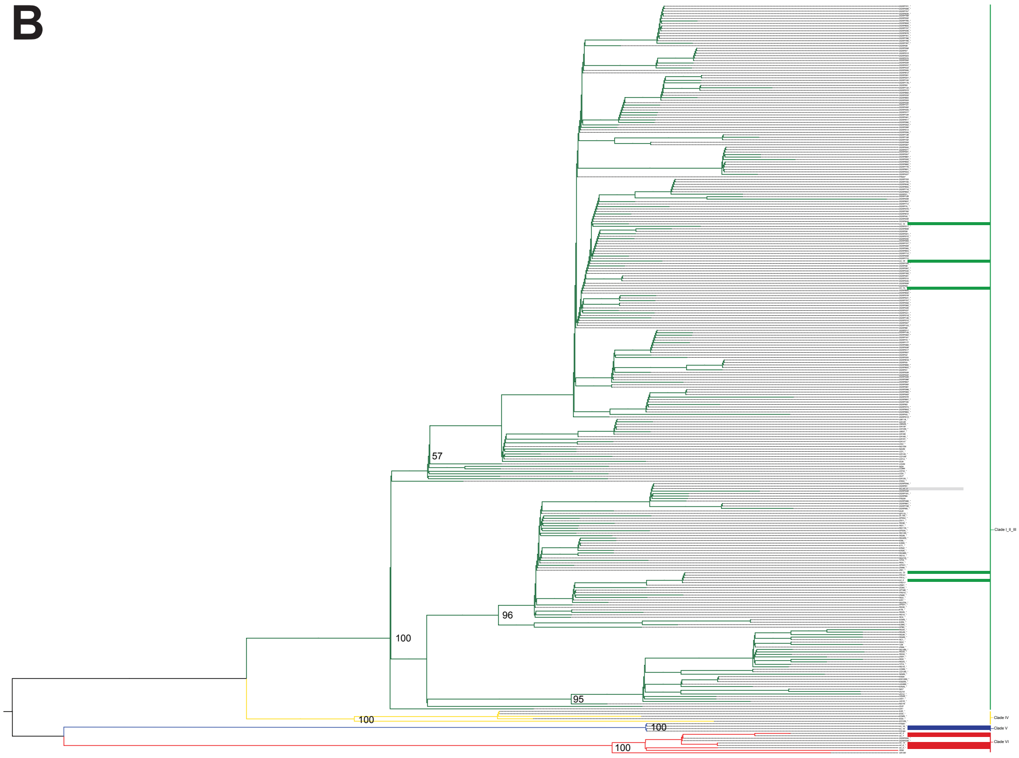

Supplement: Supplementary file 1 — Supplementary Appendix S1 Appendix S2 Appendix S3 Appendix S4 Table S1 Details about the individual flies sequenced. Table S2 Details about the populations analysed with Pool-Seq. Table S3 Influence of temperature on mtDNA and Wolbachia coverage. Fig. S1 Cumulative coverage for clade-specific SNPs. Fig. S2 Phylogenetic relationship of Wolbachia and mtDNA haplotypes. [file mec0023-0802-sd1.pdf]
